# Battlefield exoskeleton usability for lower-limb trauma in prolonged field care: A mixed-methods approach

**DOI:** 10.1017/wtc.2025.10036

**Published:** 2026-06-17

**Authors:** Ciera A. Price, Catharina C. Gaeth, Julia A. Gambill, William Brett Johnson, Sarah Pesquera, Walter Lee Childers

**Affiliations:** 1 Center for the Intrepid, Brooke Army Medical Center, USA; 2 Henry M Jackson Foundation for the Advancement of Military Medicine Inc, USA; 3 US Army Institute of Surgical Research, USA; 4 German Armed Forces Central Hospital, Koblenz, Germany; 5 Extremity Trauma and Amputation Center of Excellence, USA

**Keywords:** exoskeleton, usability, user satisfaction, fracture management, prolonged field care

## Abstract

Prolonged field care is increasingly important in military operations, where austere environments and delayed medical evacuations necessitate extended injury management. Tibia and fibula fractures are common, survivable battlefield injuries that demand such care. Current treatment options involve simple splints, which require the injured Service member to be carried on a litter, and exoskeletons, which are not suited for use in combat environments. The Intrepid Battlefield Exoskeleton (IBEX) was developed to address this critical gap in battlefield trauma care by stabilizing lower leg fractures and enabling independent mobility in prolonged care scenarios. This study evaluated the usability of two IBEX prototypes through sequential laboratory and field testing under two user conditions: medic and casualty. In laboratory testing, 20 participants completed a series of functional tasks with and without the IBEX and provided quantitative usability ratings and qualitative feedback via self-report measures and interviews. In field testing, 12 participants used the IBEX during a simulated mass casualty event and completed the same usability assessments. Quantitative results indicated high overall user satisfaction. Thematic analysis of qualitative feedback revealed recurring comments on usability, portability, comfort, security, and durability. Overall, results indicated moderate usability in both environments, with room to improve ease of fitting and adjustability. Findings highlight the value of a mixed-methods approach to device evaluation and underscore the importance of iterative design and testing. Ongoing work will compare the IBEX with the current standard of care and further refine the design for operational use.

## 1. Introduction

Prehospital trauma care in military settings presents unique challenges that are shaped by rugged terrain, extreme environmental conditions, and the demanding nature of combat missions. These factors complicate an already high-stakes operational environment where timely medical intervention is critical (Butler et al., 2017). In remote or contested regions, far from definitive care, prolonged field care becomes essential. Medics are required to manage injuries and sustain life for extended periods, often far beyond the traditional “golden hour” for casualty evacuation (Shackelford et al., 2024). As future conflicts anticipate evacuation delays stretching into hours or days, ensuring survivability in these environments remains a top priority alongside mission completion (Keenan and Riesberg, 2017).

Among the survivable injuries likely to be encountered in these scenarios, extremity fractures are common across multiple conflicts and are operationally significant, accounting for a substantial proportion of injuries sustained during deployment (Schoenfeld et al., 2013). During the Iraq and Afghanistan conflicts between 2001 and 2005, fractures were the second-most frequent injury after gunshot wounds, with the tibia being the most affected site. Notably, 82% of extremity fractures were open, requiring special consideration (Owens et al., 2007). Proper stabilization techniques, such as external splinting, are critical for reducing pain, minimizing blood loss, and preventing further soft tissue damage (Camuso, 2006; Martin et al., 2020). However, in military settings, where conditions are often austere and unpredictable, more adaptive approaches to fracture management are necessary (Butler et al., 2017). Traditional splints stabilize fractures but do not permit mobility or weight bearing, with the current standard of care being SAM® splints (SAM Medical, Tualatin, OR, USA), moldable aluminum sheets encased in foam that can be conformed to long bones for structural support (Childers et al., 2023). While commonly used, SAM® splints have been shown to permit excessive motion at the fracture site, likely compromising stabilization (Martin et al., 2020; Johnson et al., 2024a). Moreover, they render Service members (SMs) with lower limb injuries immobile, requiring litter transport. This evacuation method diverts four to six personnel for transport and up to five additional SMs for security, meaning that a single injury can significantly degrade the unit’s effectiveness (US Army, 2007).

In contrast, numerous exoskeletons enable mobility but lack stabilization components, making them contraindicated for individuals with lower limb fractures and unsuitable for prolonged field care scenarios (Johnson et al., 2023 for review). For the purpose of this paper, we adopt the ASTM International (formerly American Society for Testing and Materials) F3323-19 definition of an exoskeleton as, “a wearable device that augments, enables, assists, or enhances motion, posture, or physical activity” (Lowe et al., 2019). Existing exoskeletons for industrial and military applications primarily aim to enhance human performance by increasing load-carrying capacity or reducing metabolic cost, while medical use exoskeletons support stability and mobility during physical therapy or home use (Lowe et al., 2019; Johnson et al., 2023). However, these devices are often too bulky, heavy, or fragile for effective deployment in combat environments (Franklin et al., 2019; Johnson et al., 2023). Taken together, splints provide stabilization without mobility, while exoskeletons offer mobility without stabilization, leaving a critical capability gap in fracture management during prolonged field care. A promising solution is therefore an exoskeleton that stabilizes lower limb fractures (Johnson and Childers, 2025) *and* redirects the ground reaction forces around the injury to restore independent mobility in austere environments (Childers et al., 2024).

The Intrepid Battlefield Exoskeleton (IBEX) was specifically engineered to meet this need. The IBEX is a passive exoskeleton designed to stabilize and offload lower leg injuries, including open tibia/fibula fractures, enabling SMs to independently navigate diverse and challenging terrains (e.g., mud, sand, snow, and steep inclines). It is not specific to left or right, fits approximately 90% of the US Army, is portable, and weighs approximately 3 kg. The design incorporates a splinting component to stabilize the fracture and offloading components that redistribute weight to an external frame, enabling independent mobility. The IBEX is intended to serve as an alternative to litter-based casualty evacuation, but little is known about its usability in operational settings. This mixed-methods study examines the usability of two IBEX prototypes (Mark I and Mark II) and evaluates the design for field use. By understanding how usability testing can inform design changes, this research aims to improve battlefield fracture management and support the self-rescue capabilities of wounded SMs in prolonged care scenarios. This usability testing and the resulting exoskeleton design may also be applicable to wilderness care and other nonmilitary remote rescue situations.

## 2. Methods

The following methods describe two sequential experiments: laboratory testing of the Mark I to evaluate initial exoskeleton performance and safety, followed by field testing of the Mark II to assess usability and functionality during simulated military operations. Usability testing captured quantitative and qualitative input from participants in two operational roles: medics, who apply the exoskeleton as an intervention, and casualties, who utilize the exoskeleton as a mobility aid. Both perspectives are essential as challenges in transport and application by the medic or comfort and safety of the casualty would limit feasibility in field settings.

### 2.1. Laboratory testing

#### 2.1.1. Participants

Participants included able-bodied adults (18–40 years) who were authorized to receive care at Brooke Army Medical Center (BAMC) and were able to comply with testing instructions. Participants with self-reported lower extremity musculoskeletal injuries, skin irritation, or pregnancy were excluded. As the design accommodated individuals between the 5th percentile female and 95th percentile male SM based on anthropometric data (Gordon et al., 2014), participants shorter than 1.52 m, taller than 1.85 m, or weighing more than 108.9 kg were ineligible for participation.

#### 2.1.2. Exoskeleton design

**Figure 1.**
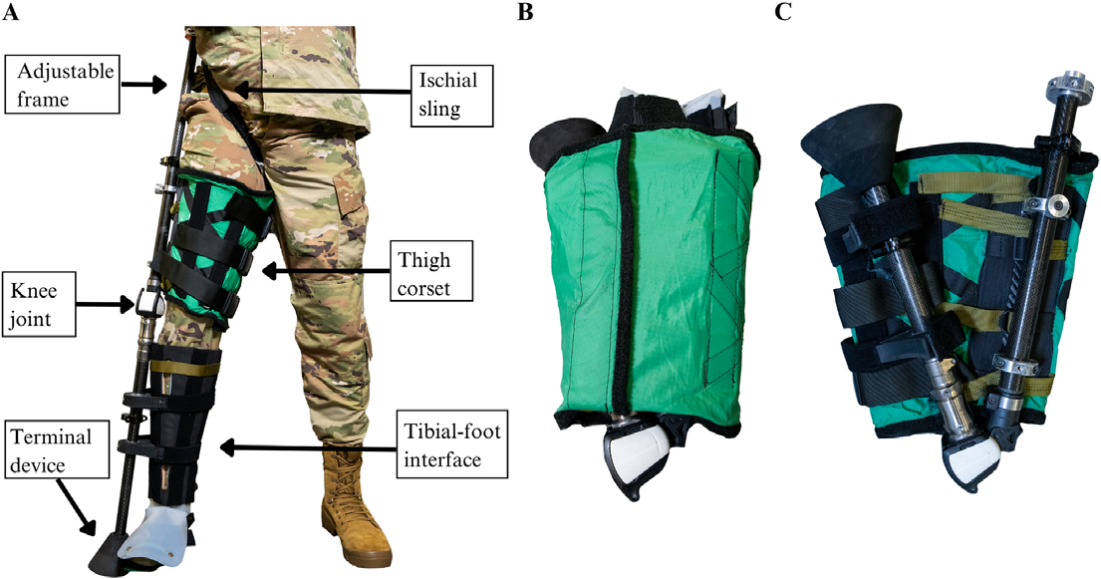
The Mark I (mass = 2.84 kg, pack volume = 23.3 L) with key design elements highlighted. (a) Anterior view of the Mark I fit to a Service member. (b) The Mark I wrapped in its thigh corset in a fully folded and collapsed configuration. (c) The Mark I unwrapped, demonstrating the 30° folding angle of the knee joint. Figure 1. long description.

The IBEX Mark I (Figure 1(a and b)) featured a telescopic frame, offloading components, knee joint, tibial–foot interface, and terminal device. This design was informed by clinical knowledge of offloading principles, as applied in orthotics and prosthetics management, and practical knowledge gleaned from our Tactical Medical Advisory Panel comprised of Army Combat Medics and other field medicine practitioners (Johnson et al., 2024b). The Mark I frame incorporated two pairs of carbon fiber tubes, each consisting of an inner and outer tube, creating a height-adjustable frame. The length of the frame could be adjusted via cam-actuated clamps (e.g., bicycle clamps) fitted to the outer tubes. The offloading components consisted of an ischial sling and thigh corset, both attached to the proximal aspect of the frame and designed to facilitate weight bearing through the pelvis and proximal thigh, respectively. This combination of offloading components was previously tested and shown to minimize relative motion between the frame and the user (Johnson et al., 2025). The ischial sling, made of polyester and cotton, was fit through the groin to support the posterior distal aspect of the pelvis and was secured with hook and pile fasteners. The thigh corset, made of ripstop nylon and reinforced with aluminum stays positioned parallel to the femur, wrapped circumferentially around the thigh. The corset was rigidly attached to the frame via a laterally placed carbon fiber plate and secured with polyester straps and parachute buckles. A single-axis prosthetic knee joint (Modular Knee Joint, Ottobock, Austin, TX, USA) allowed the proximal and distal sections of the adjustable frame to fold from a fully extended position to 30° (Figure 1(c) **)**. The tibial–foot interface was constructed similarly to the thigh corset but wrapped around the lower limb to stabilize the fracture site, and included a thin, conformable plastic sheet to support the foot–ankle joint. Foam spacers were positioned between the interface and distal frame where hook and pile fasteners secured the spacers to the injured limb. These spacers were able to slide freely along the frame, preventing any vertical displacement between the frame and the user from exacerbating the injury. The terminal device was inspired by the All-Terrain prosthetic foot and constructed from a rubber-like foam (Matthews et al., 1993). This component was adhered to the distal frame and extended beyond the anatomical foot to allow ground contact with the frame rather than the injured limb.

#### 2.1.3. Test protocol

The study protocol (C.2023.003) was approved by the San Antonio Institutional Review Board (IRB) in compliance with applicable Federal regulations governing the protection of human subjects. Participants were recruited via approved flyers distributed throughout BAMC, and written informed consent was obtained from all participants before initiating any study procedures.

Two in-lab visits were conducted to evaluate the performance and usability of the Mark I under two user conditions: (1) as a Medic (who applies the exoskeleton to a simulated casualty) and (2) as a casualty (who wears the exoskeleton). All participants (*n* = 20) completed both user conditions. During each visit, participants were provided form-fitting workout clothes and fit with a standard issue helmet (1.3 kg), vest (1.1 kg), and a mock M16 rifle (3.2 kg). Participants wore their own athletic shoes. Participants were provided standardized instructions and completed a series of functional and military-specific tasks designed to evaluate mobility. The following tasks were completed in a randomized order: (1) overground walking at a self-selected speed over a 10 m walkway, (2) weaving through a series of cones while carrying two ammunition cans (9 kg each), (3) transitioning between standing, kneeling, and prone positions to engage and fire at a stationary target, and (4) the Timed-Up and Go (TUG) test. The TUG test is a clinical mobility measure requiring participants to rise from a chair, walk three meters, turn 180°, return to the chair, and sit down (Podsiadlo and Richardson, 1991). During the first visit, participants completed the medic condition, performing all tasks without the Mark I and then applying the exoskeleton to study personnel following a fitting demonstration. During the second visit, participants completed the casualty condition, performing all tasks with the Mark I. At the conclusion of each visit, participants completed two self-report surveys (described subsequently) and an informal interview from the perspective of either the medic or casualty. Additionally, during both visits, kinetic, kinematic, and electromyography data were recorded as part of a larger study.

#### 2.1.4. Outcome measures

Participants completed the System Usability Scale (SUS) and the Quebec User Evaluation of Satisfaction with Assistive Technology (QUEST) under both user conditions. The SUS requires users to rate statements about the system on a five-point Likert scale from 1 (strongly disagree) to 5 (strongly agree) and produces a composite score that provides insight into the overall usability of the system (Brooke, 1996). The QUEST measures user satisfaction on a five-point Likert scale from 1 (not satisfied at all) to 5 (very satisfied) across eight subscales: dimensions, weight, ease in adjusting, safety, durability, ease of use, comfort, and effectiveness. The subscale scores are averaged to produce an overall satisfaction score. The QUEST also requires users to rate three of the subscales as “most important” (Demers et al., 1996). Following the self-report surveys, study personnel asked participants open-ended questions about the usability, feasibility, and possible design improvements of the Mark I.

### 2.2. Field testing

#### 2.2.1. Participants

Participants included active-duty SMs from the US Army Alpha Company, 1–29 Infantry; US Army 2nd Battalion, 2nd Infantry Regiment; British Army Alma Company, 2nd Battalion, The Royal Yorkshire Regiment; Dutch Army 17th Armoured Infantry Battalion, Bravo Company; and German Army Versuchskräfte Heer.

#### 2.2.2. Exoskeleton design

**Figure 2.**
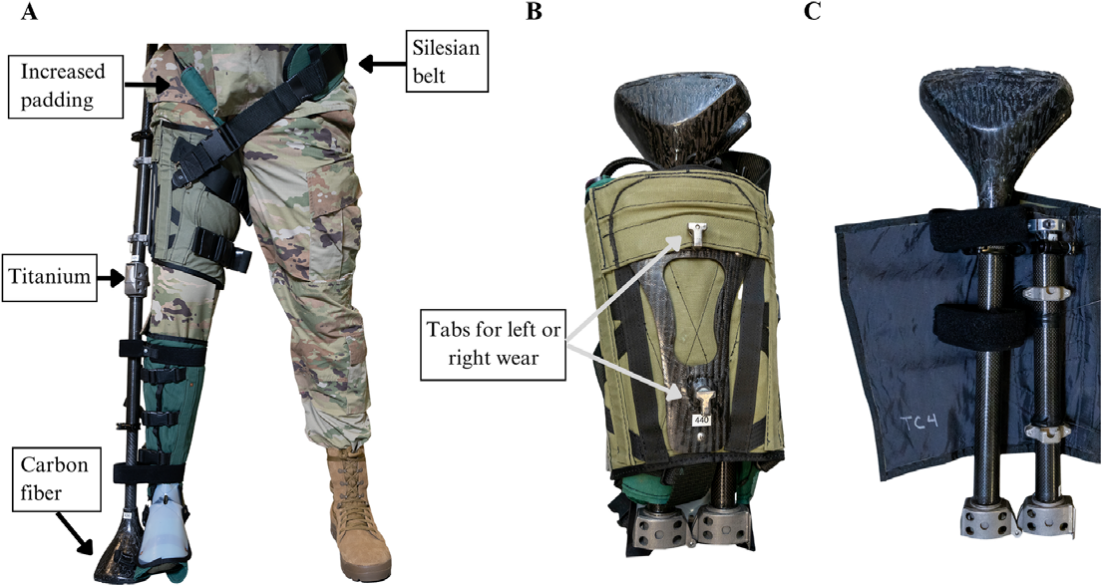
The Mark II (mass = 2.95 kg, pack volume = 22.5 L) with key design improvements is highlighted. (a) Anterior view of the Mark II fit to a Service member. (b) The Mark II wrapped in its thigh corset in a fully folded and collapsed configuration. (c) The Mark II unwrapped, illustrating the enhanced compactability of the frame. Figure 2. long description.

The IBEX Mark II (Figure 2(a)) featured several critical improvements relative to the Mark I used in laboratory testing. The ischial sling, comprising a wide cotton pad and polyester strap, was replaced with a cylindrical foam pad and nylon strap for improved comfort. The sling extended posteriorly and wrapped around the contralateral pelvis to provide enhanced suspension and security, similar to the Silesian belts used in conventional prosthetic management (Muller, 2016). Custom titanium collars with left and right slots were fit to the proximal frame and the thigh corset was modified with T-shaped tabs that fit into either slot (Figure 2(b)). This modification enables the exoskeleton to be quickly configured for either the left or right leg, enhancing field utility by eliminating the need to transport multiple devices. The commercial prosthetic knee was replaced with a custom, 3-D printed titanium knee (Figure 2(c)) with decreased weight and increased range of motion, allowing the frame to fold completely in half for improved portability. Finally, the dense foam used in the terminal device was replaced with lightweight carbon fiber, resulting in reduced mass and increased durability.

#### 2.2.3. Test protocol

This protocol was deemed not research by the San Antonio IRB, and thus obtaining informed consent was not required. Demographic information was not collected due to the nature of the military exercise, which limited interactions with the SMs using the exoskeleton.

The Mark II was tested during the 2024 Army Expeditionary Warrior Experiment (AEWE) hosted by the Maneuver Battle Laboratory in Fort Moore, GA over a two-day period. The AEWE is an annual event that provides an opportunity to test new technologies during simulated military missions, with the end goal of supporting combat readiness. The Mark II was tested alongside other casualty care technologies (e.g., an upper extremity harness to support litter carries) to treat tibia fractures, gunshot wounds, or facial wounds. On Day 1, SMs received training for each of the technologies to be evaluated. Lab personnel led a 1-hr training session where SMs received a 15-min demonstration on how to apply the Mark II to a simulated casualty with a tibia fracture. SMs used the remaining 45 min of the training session to practice Mark II application and receive feedback. On day 2, a total of four Medical Stakes missions were conducted in an outdoor, all-terrain environment. The objective of the Medical Stakes mission was to assess, treat, and evacuate simulated casualties following a mass casualty scenario. For each mission, two SMs received mission instructions and a Mark II, then moved tactically to a casualty collection point where they triaged injured SMs, identified the casualty with the tibia fracture, applied the Mark II, and evacuated the casualty to a helicopter loading zone 80–120 m away. In total, 12 SMs used the Mark II (*n* = 8 as medics; *n* = 4 as casualties). At the conclusion of the mission, all SMs completed the previously described self-report surveys from the perspective of either a medic or casualty depending on the role played during the simulation. No interviews could be conducted.

#### 2.2.4. Outcome measures

Participants completed the SUS and QUEST surveys to assess usability.

### 2.3. Statistical analysis

Quantitative survey data were descriptively analyzed using summary statistics (e.g., means, standard deviations) to characterize participant responses. All survey and interview comments were qualitatively assessed via a general inductive approach in which broad themes were established from the collected data (Thomas, 2006). Comments were iteratively coded, and themes were revised as additional participants completed the protocol. Coding consistency was assessed via independent parallel coding. Two investigators independently coded the data, and their analyses were compared to assess overlap. There was further discussion in cases of low overlap and the identified themes and subthemes were merged.

## 3. Results

### 3.1. Laboratory testing

Twenty participants were consented into the study and completed both user conditions during the laboratory testing protocol; demographic information is provided in Table 1.

**Table 1.** Participant demographic information Table 1. long description.

Participant	Gender	Age (y)	Height (m)	Mass^a^ (kg)	Race	Branch	MOS
01	F	28	1.52	56.0	Asian	N/A	Civilian
02	M	35	1.80	96.1	White	Army	Health Care Specialist
03	M	32	1.73	90.7	White	Army	Occupational Therapy Specialist
04	M	25	1.85	102.7	Asian	Army	Medical Laboratory Specialist
05	M	29	1.83	90.9	AI/AN	Army	Medical Laboratory Specialist
06	M	25	1.60	72.1	White	Air Force	Clinical Nurse
07	M	30	1.78	83.0	White	Army	Health Care Specialist
08	M	30	1.83	112.0	White	Air Force	General Practice Physician
09	F	33	1.63	81.2	White	Navy	Orthopedic Cast Room Technician
10	M	26	1.78	92.3	White	Navy	Hospital Corpsman
11	M	36	1.80	114.8	Asian	Air Force	Physical Therapist
12	M	28	1.78	93.0	White	Army	Animal Care Specialist
13	F	26	1.55	67.8	White/Black	Army	Animal Care Specialist
14	M	34	1.83	103.1	White	Army	Health Care Specialist
15	F	30	1.65	67.4	White	Army	Health Care Specialist
16	M	39	1.68	83.3	Asian	Army	Health Care Specialist
17	M	36	1.85	98.7	White	Army	Health Care Specialist
18	M	37	1.80	89.1	White	Army	Health Care Specialist
19	M	34	1.63	78.8	White	Army	Health Care Specialist
20	M	26	1.80	84.6	White	Air Force	Medical Service Specialist
Median	—	30	1.78	89.9	—	—	—
Max	—	39	1.85	114.8	—	—	—
Min	—	25	1.52	56.0	—	—	—

Abbreviations: AI/AN, American Indian/Alaska Native; MOS, Military Occupational Specialty.

a Mass was recorded during Visit 1 with the fitted vest and helmet.

#### 3.1.1. Usability and satisfaction scores

**Figure 3.**
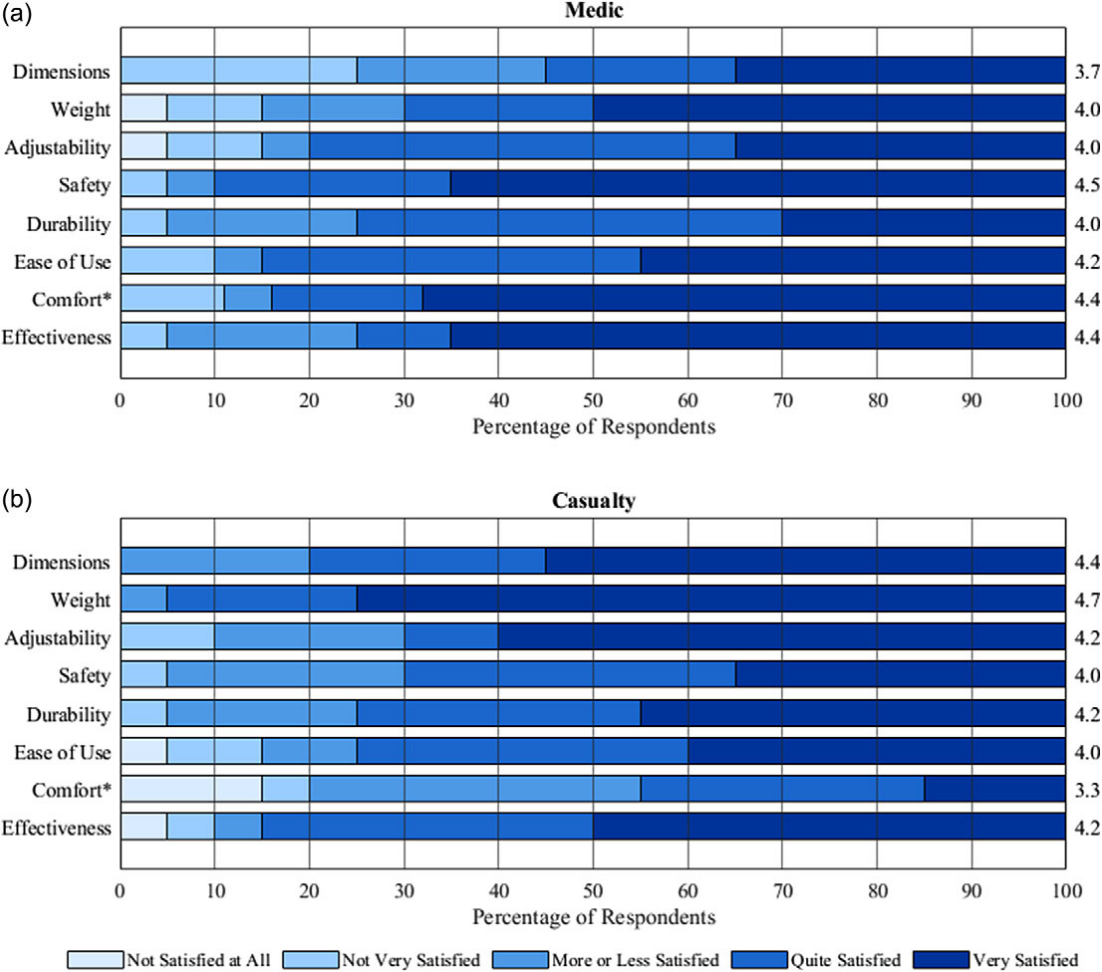
Distribution of the QUEST subscale satisfaction scores during laboratory testing of the Mark I in the (a) medic and (b) casualty conditions. Values on the right *y*-axis represent the mean subscale scores. *Results shown for the comfort subscale are for *n* = 19 participants due to one incomplete response. Figure 3. long description.

The median SUS composite score was 78.8 (IQR = 70.0–90.6) for the medic condition and 72.5 (IQR = 65.0–86.3) for the casualty condition. The mean QUEST overall satisfaction score was 4.1 or “quite satisfied” from both the medic (SD = 0.82) and casualty (SD = 0.66) perspectives. Subscale analysis (Figure 3) revealed that participants were least satisfied with dimensions and weight in the medic condition, and comfort in the casualty condition. Effectiveness was universally rated as the most important subscale and had mean satisfaction scores of 4.4 and 4.2 for the medic (SD = 1.0) and casualty (SD = 1.1) conditions, respectively.

#### 3.1.2. Qualitative analysis

**Figure 4.**
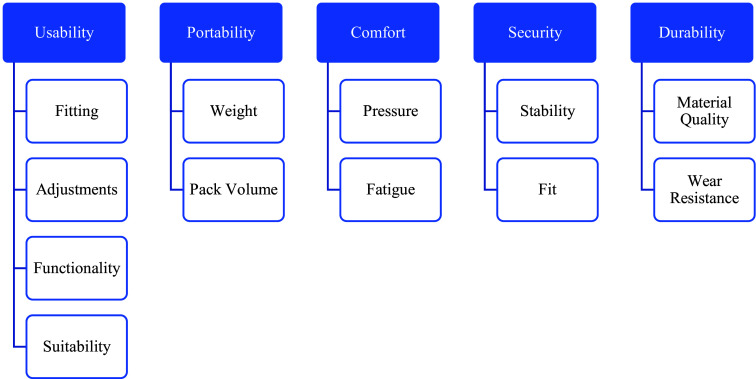
Thematic analysis identified five distinct themes with two to four subthemes in each.

A total of 324 participant comments were included in the qualitative analysis resulting in five themes: usability, portability, comfort, security, and durability (Figure 4). A range of comments, both positive and negative, are presented below to guide a comprehensive discussion. The complete list of participant comments is given in the Supplementary Materials.

##### 3.1.2.1. Usability

This theme encompassed nearly 40% of the participant comments and relates to both ease of use and functionality. Subthemes include (1) the ability of the medic to intuitively fit the Mark I to a casualty, (2) ease of adjustments, (3) exoskeleton performance and effectiveness, and (4) suitability for military operations. Participants in the medic condition expressed concerns that the Mark I was complicated but could be fit appropriately given proper instruction and time to practice.

It’s a little complicated, not bad with training … It was a little frustrating the first time, with multiple reps, it’s probably easy.” (P03, Medic)

First time trying was simple, just minor practice could help. Nonresponsive patient could be difficult. (P10, Medic)

Device looks intimidating. [There’s] too much going on. (P12, Medic)

Casualty feedback varied regarding the ability to make adjustments independently. Several participants noted the need for multiple adjustments or anticipated requiring assistance, particularly with components like the terminal device. While the Mark I was designed for medics to fit to casualties, allowing users to adjust the exoskeleton for their own comfort could be advantageous and improve usability in the field.

Adjusting length at bottom end will require a partner. (P06, Casualty)

While it is easy to set up and adjust, it requires multiple height adjustments when first placed to ensure off-loading of the leg. (P09, Casualty)

It is doable to adjust by myself. However, it feels like it needs extra hands to secure. (P16, Casualty)

Meanwhile, participants in the medic condition offered practical suggestions to improve usability, suggesting thoughtful buckle placement, consistent fasteners, and numbered or color-coded straps to improve fitting speed and accuracy. Medics also acknowledged that the ease of adjustments could vary in different environments (e.g., rain, mud, sand) and highlighted that a simplified strapping system would be beneficial in high-stress situations.

Consistent fasteners would be helpful when panicking. (P07, Medic)

Easy to adjust in current environment. Sliding [straps] with mud or sand/rust could be hard. (P10, Medic)

Fairly easy to apply after 1 demo… Printed instructions on sleeve would be helpful and color-coded straps. (P19, Medic)

Additional usability comments centered on exoskeleton performance and general usefulness for completing military tasks. Casualty feedback was mixed, with some expressing confidence in walking and running with little need for an acclimation period, while others reported difficulty completing tasks beyond basic ambulation.

It was able to help me with basic walking, but did not help that well with more advanced activities (e.g., ammo carry, prone shooting). (P01, Casualty)

Felt like I could run on it. (P04, Casualty)

You get used to it fast. [I was] surprised by how quickly I began to trust device would offload tibia. (P08, Casualty)

There is a pretty extreme learning curve to use the device to walk and perform military-relevant tasks. (P09, Casualty)

In less than 10 min I felt confident navigating around obstacles. (P19, Casualty)

Participants in both conditions generally perceived the Mark I as being useful in special forces situations, particularly for small units, but cautioned that the current closure system could result in excessive manipulation of the injured limb. Several participants noted that use of the Mark I would allow the casualty to continue supporting the unit in some capacity while one participant recommended specific roles that could be suitable.

Product has potential, but T2 [tibia–foot interface] requires over manipulation of injured limb by velcroing in back. (P02, Medic)

Recommended roles for IBEX users: could help carry equipment, run security at CCP [Contingency Command Post], radio, litter carry team on an easy terrain…Discouraged roles for IBEX users: not a point man, not a breecher [sic], support by fire, but not assault. (P14, Casualty)

It will give hand[s] free capability to casualty who can assist team. (P16, Casualty)

##### 3.1.2.2. Portability

This theme described the ability or willingness of the SM to transport the Mark I, with most comments provided during the medic condition. Subthemes were related to (1) weight and (2) pack volume. Participants were generally satisfied with the weight but suggested it could be reduced, considering the additional supplies medics are already responsible for carrying. While weight may not have been a major issue, there was a clear consensus that the Mark I was too bulky, with several participants recommending that it be collapsible or completely foldable.

Would like to fold up parallel, not at 30-degree angle; would like better telescopes to make it less long. (P01, Medic)

Weight is light; bulk is a problem; more compact would be good; has value on MEDEVAC vehicle as less space than litter. (P04, Medic)

While not excessively heavy, adding this device to all medical supplies that are required to be carried could be strenuous for the medic. (P09, Medic)

Too big to carry into field; would keep on truck. If it could fold completely, I would carry into field. (P14, Medic)

Not heavy at all … Awkward size to carry; better if pylons ran parallel. (P20, Medic)

##### 3.1.2.3. Comfort

This theme discussed the ability of the injured SM to wear and utilize the Mark I without pain or skin breakdown. Subthemes involved (1) pressure due to weight-bearing components and (2) muscle fatigue related to compensatory movements. As the exoskeleton transfers weight-bearing from the injured limb to the pelvis, participants in the casualty condition generally agreed that thicker and wider padding on the ischial sling would improve comfort. There was mixed feedback about the comfort of the thigh plate, the secondary weight-bearing component, with some participants expressing that the edges of the plate irritated the soft tissues of the thigh. Additionally, participants noted the exoskeleton prevents the injured limb from contacting the ground, creating a leg length discrepancy that requires a change in gait – these compensatory movements caused some muscle fatigue in the back and unaffected hip.

“Pelvic area support was focused on a small area of pressure. Length of device and lack of offset on the other leg put lots of pressure on the non-device leg.” (P01, Casualty)

“Suggests thicker padding on ischial strap and wider support.” (P07, Casualty)

“A lot of weight transitioned to groin, more padding requested, wanted to be more even for leg lengths…Extra padding on the ischial sling would increase comfort.” (P08, Casualty)

“Mild to moderate discomfort in the groin area and at the top of the thigh plate. Compensation created muscle fatigue.” (P09, Casualty)

##### 3.1.2.4. Security

This theme related to the ability of the Mark I to operate safely and reliably, ensuring sufficient stability for ambulation and other dynamic activities. Subthemes involved (1) general safety and stability and (2) exoskeleton fit. Participants’ confidence in security varied, with several highlighting that exoskeleton fit, particularly the ability to adjust straps and other components to ensure a tight fit, contributes to overall safety when navigating diverse terrains.

It seems very secure once on. (P10, Medic)

Felt like device wasn’t secure to my body; shifted a lot while walking. (P12, Casualty)

Moderately secure, can see possible safety issues with straps…Terminal device should be metal, more solid, not wobbly on rubble. (P15, Medic)

If securing strap comes [in] different sizes, [then] device can be secured more. (P16, Casualty)

##### 3.1.2.5. Durability

The final theme describes the ability of the overall exoskeleton, or any individual component, to withstand normal use over an extended period. Subthemes involved (1) material quality and (2) wear resistance. Participants perceived that the Mark I, designed as a single-use device, could support ambulation for up to 1 week, but highlighted that certain components, particularly the hook-and-pile straps and plastic buckles, could be replaced with stronger materials to improve durability.

Velcro in all weather conditions? (P06, Medic)

Prefers metal buckles for durability; concerned plastic buckles will break during transport and storage. (P15, Casualty)

How easy is it going to be to get replacement part? Non-metal parts can be worn out or broken. (P16, Casualty)

Depending on if it is single use or not, could see Velcro and fabric wearing out but carbon fiber and metal pieces I would have no worries with. (P20, Medic)

### 3.2. Field testing

**Figure 5.**
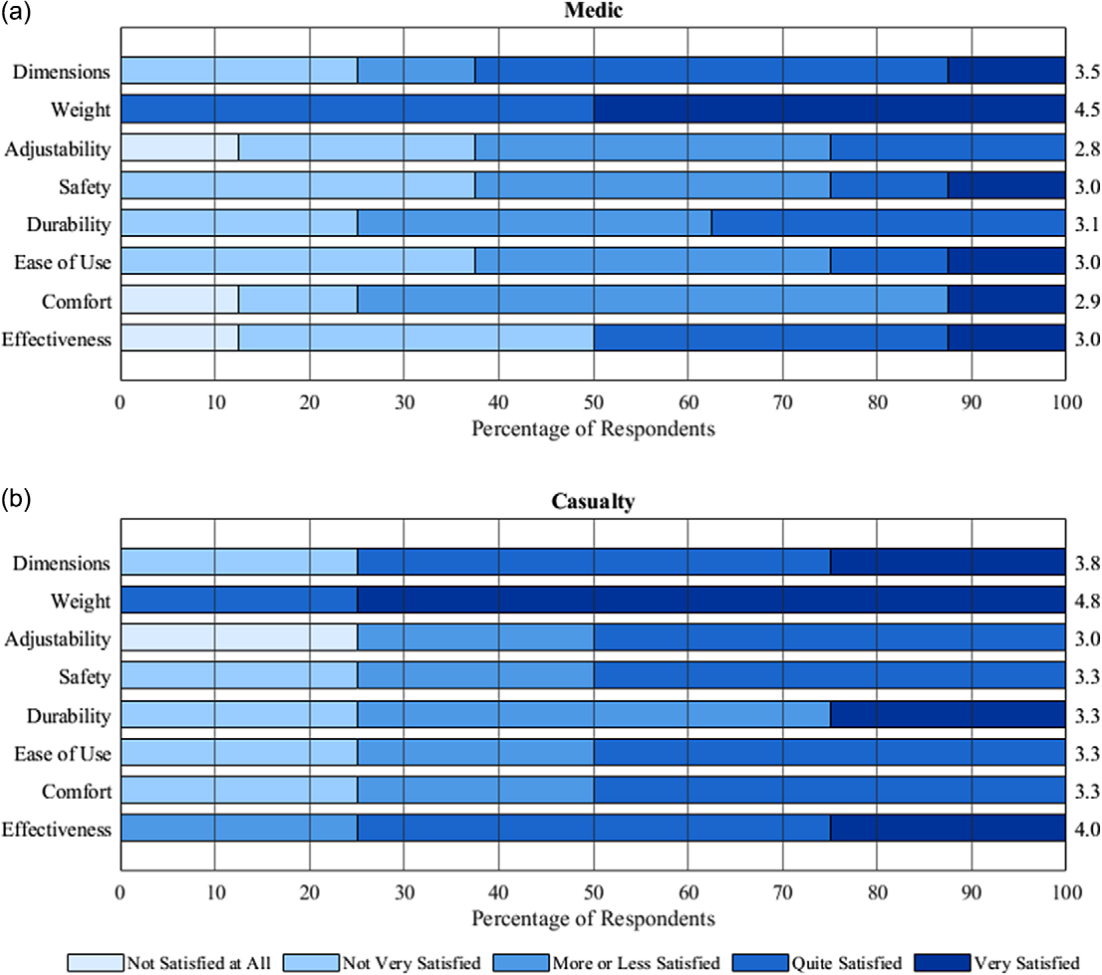
Distribution of the QUEST subscale satisfaction scores during field testing of the Mark II in the (a) medic and (b) casualty conditions. Values on the right *y*-axis represent the mean subscale scores. Figure 5. long description.

Twelve participants completed field testing with eight participants in the medic condition and four participants in the casualty condition. The median SUS composite score was 67.5 (IQR = 59.4–74.4) for the medic condition and 65.0 (IQR = 54.4–70.6) for the casualty condition. The mean QUEST overall satisfaction score was 3.6 ± 0.8 and 3.2 ± 0.7 in the medic and casualty conditions, respectively. Subscale analysis (Figure 5) revealed that participants in both conditions were least satisfied with the ease of adjustments and most satisfied with the weight. In the medic condition, participants rated effectiveness as the single-most important subscale and were “more or less satisfied” with a mean score of 3.0 ± 1.4. In the casualty condition, weight and durability tied as the most important subscale with mean satisfaction scores of 4.8 ± 0.5 and 3.3 ± 1.3, respectively. Qualitative analysis was not performed as the field test data did not produce sufficient participant comments for thematic review.

## 4. Discussion

### 4.1. Laboratory testing

The mixed-methods approach, incorporating both survey and interview data, provided quantitative feedback on system usability and satisfaction, as well as rich insights on potential exoskeleton improvements. The SUS is a widely used outcome measure with well-established benchmarks for interpreting usability (Hajesmaeel-Gohari et al., 2022). The Mark I met the SUS acceptability threshold of 68.0 in both user conditions (Bangor et al., 2009). The medic score of 78.8 corresponds to a product usability rating of “good” to “excellent,” while the casualty score of 72.5 is interpreted as “good” (Bangor et al., 2009; Brooke, 2013).

The QUEST provides detailed information about user experience across eight subscales (Demers et al., 2002). The mean overall satisfaction score was a 4.1 for both the medic and casualty conditions, corresponding to “quite satisfied.” Subscale analysis revealed important distinctions between the two user conditions. In the medic condition (Figure 3(a)), participants reported moderate satisfaction with Mark I dimensions (3.7 ± 1.2) and weight (4.0 ± 1.3). Although the average scores suggest general satisfaction, these domains had the fewest ratings in the “quite satisfied” and “very satisfied” categories, highlighting areas for potential improvement. In contrast, participants in the casualty condition (Figure 3(b)) reported the highest satisfaction with both dimensions (4.4 ± 0.8) and weight (4.7 ± 0.6), with 80% of participants rating dimensions and 95% rating weight as “quite satisfied” or “very satisfied.” Comfort received the lowest score for casualties (3.3 ± 1.3), with participants noting three areas for improvement: increasing padding and support on the ischial sling, reducing irritation from the thigh plate edges, and addressing the leg length discrepancy due to limb offloading. These differences likely reflect role-specific priorities: medics must transport the device and therefore prioritize weight and pack volume, whereas casualties wear the device to restore mobility and may prioritize comfort.

Qualitative feedback was highly informative for the Mark II design. Although the Mark I (2.84 kg) was not perceived as heavy in isolation, participants indicated that reducing bulk and weight would benefit medics already carrying substantial loads. Suggestions for improved foldability and pack integration motivated changes to the knee design. Casualty feedback on comfort and security, including confidence in dynamic tasks and strapping effectiveness, led to adjustments in the padding and suspension systems.

It is difficult to interpret these results in context due to the limited literature on exoskeleton usability for acute lower-limb injury in military settings. Usability studies of exoskeletons for spinal cord injury in home or therapy use exist (van Dijsseldonk et al., 2020; Basla et al., 2022; Rodríguez-Fernández et al., 2025), but comparisons are challenging given the different patient populations and use cases. While literature exists on exoskeletons for military applications, these are either not designed for lower limb use (Slaughter et al., 2023) or usability outcomes are not reported (Yu et al., 2014; Collins et al., 2015; Quinto et al., 2025). However, the US Army has outlined design considerations for exoskeletons intended for casualty evacuation, emphasizing the need for lightweight, portable, one-size-fits-most devices that are comfortable, durable, safe, reliable, and easy to use (Madison et al., 2022). Our choice of outcome measures directly assessed these qualities, and the results suggest that while the Mark I met several of these criteria under laboratory conditions, there remains room for improvement.

### 4.2. Field testing

Based on laboratory feedback, the Mark II incorporated several modifications, including increased padding for comfort, a Silesian belt for added security, T-shaped tabs for left or right wear, a titanium knee joint to reduce pack volume and improve portability, and a more durable, lightweight terminal device. These changes were expected to improve usability and satisfaction, yet SUS and QUEST scores decreased in both user conditions during field testing, indicating that satisfaction varied across scenarios and further refinements are necessary. One exception was weight, where QUEST scores improved from 4.0 to 4.5 in the medic condition and from 4.7 to 4.8 in the casualty condition. Other design improvements, however, were not reflected in subscale scores. The lower scores in field testing likely reflect the transition from a controlled environment to simulated combat missions involving longer distances, uneven terrain, distracting sounds, and pressure to work quickly. These conditions more closely mirror operational use, particularly with respect to physical demands and endurance. Reduced satisfaction may also reflect limited user acclimatization to the Mark II and the added challenges of prolonged use in combat-like conditions. While it is uncertain whether extended laboratory testing would have demonstrated benefits, field testing clearly identified real-world challenges and tactical requirements that could not be fully replicated in controlled settings.

### 4.3. Limitations

The sample size was relatively small, particularly within the field-testing cohort, and predominately male, which may limit the generalizability of findings, as user needs and expectations can vary across demographic groups. Selection bias may have influenced the laboratory test results as SMs with high interest in exoskeleton development volunteered to participate, potentially affecting the findings. Additionally, the SUS and QUEST surveys provided limited depth due to their standardized nature and emphasis on specific usability characteristics that may not have had enough specificity for a battlefield exoskeleton and may have neglected certain aspects of the exoskeleton’s performance. The qualitative interviews complemented survey results, but the questions were not uniformly structured, and not all participants provided equivalent feedback, complicating data aggregation and the derivation of consistent conclusions across the entire sample. Finally, the authors acknowledge that the results from laboratory and field testing are not directly comparable, and neither environment can fully replicate the battlefield. Laboratory testing employed the Mark I, which lacked certain enhancements implemented for field testing. This complicates direct result comparisons and suggests that any improvements observed in the field cannot be solely attributed to design modifications. Furthermore, while field testing offered more realistic conditions compared to laboratory testing, the settings remained somewhat controlled; casualties were still simulated; and external factors, such as pain, polytrauma, and the high-stress nature of combat, were not fully addressed. Consequently, the results may not fully encapsulate all the challenges the exoskeleton would encounter in a real-world, prolonged care scenario.

### 4.4. Future work

To address these limitations and further improve the IBEX design, continued data analysis and exoskeleton development will take place in parallel with additional field testing. First, laboratory testing of the Mark I was part of a larger data collection that may help to define exoskeleton performance and loading requirements during military-specific tasks. Second, feedback from the laboratory and field tests described here have already informed Mark III development. Several improvements have been implemented, including printed instructions to enhance ease of use, a 63% reduction in pack volume compared to the Mark I, and design changes that reduce the leg length discrepancy for improved effectiveness and comfort. Finally, future work will involve field testing the Mark III with larger sample sizes against the current standard of care (i.e., application of a SAM® splint and use of a litter team). This work will incorporate both subjective survey data and objective performance metrics, including heart rate data and task completion time, and participants will navigate over 1 km of outdoor, uneven terrain.

## 5. Conclusion

Innovative solutions are needed to address gaps in combat casualty care and fracture management given the anticipated increase in evacuation times for future conflicts. Existing technologies are often ill suited for austere environments or stabilize fractures in a manner that necessitates a litter carry. The current study demonstrated good to excellent usability of a battlefield exoskeleton, designed to offload the injured limb and enable independent mobility, when tested during simulated military operations. During laboratory testing, participants in both user conditions reported satisfaction with exoskeleton effectiveness and safety. Qualitative results revealed positive feedback on perceived exoskeleton usefulness, particularly for enabling casualties to serve in support roles, thereby enhancing whole unit effectiveness. Field testing indicated moderate usability, highlighting the need to prioritize adjustability and ease of use in future development. Overall, results suggest that the Mark II, while theoretically improved from the Mark I, is not yet ready for field deployment, underscoring the importance of testing battlefield technologies in more realistic environments. This mixed-methods study highlights the value of qualitative research in shaping user-centered solutions and illustrates how iterative development and testing can inform design improvements. While ongoing work will continue to refine the IBEX, battlefield exoskeletons hold considerable potential to redefine what is possible in prolonged field care.

## Supporting information

10.1017/wtc.2025.10036.sm001 Price et al. supplementary material Price et al. supplementary material

## Data Availability

The data underlying this article will be shared on reasonable request to the corresponding author and appropriate clearances gained to maintain operational security.

## Supplementary material

The supplementary material for this article can be found at http://doi.org/10.1017/wtc.2025.10036.

## Figure 1. Long description

Panel A at left shows a Service member wearing the Mark I exoskeleton on the right leg. From top to bottom, labeled components are: adjustable frame alongside the leg, ischial sling at the pelvis, thigh corset wrapping the upper leg, knee joint at the knee, tibial-foot interface at the lower leg, and terminal device extending beyond the foot. Panel B at center displays the Mark I fully folded and collapsed, wrapped in its green thigh corset, with the terminal device and tibial-foot interface visible at the top. Panel C at right shows the Mark I unwrapped, with the knee joint bent at a 30 degree angle. The green thigh corset is open, exposing the internal structure and mechanical components.

Navigate back to Figure 1..

## Figure 2. Long description

Panel A on the left shows an anterior view of a service member wearing the Mark II exoskeleton on the right leg. Labeled callouts identify increased padding at the thigh, a Silesian belt at the waist, a titanium knee, and carbon fiber terminal device. Panel B in the center displays the Mark II fully folded and wrapped in a green thigh corset, with arrows pointing to tabs for left or right wear. Panel C on the right presents the Mark II unwrapped, revealing the compact frame with visible carbon fiber and titanium rods, and Velcro straps securing the assembly. The sequence demonstrates the exoskeleton's modularity and enhanced portability.

Navigate back to Figure 2..

## Table 1. Long description

The table contains eight columns: Participant, Gender, Age in years, Height in meters, Mass in kilograms, Race, Branch, and MOS. Rows 1 to 20 list individual participants. For example, participant 01 is female, age 28, height 1.52 meters, mass 56.0 kilograms, Asian, branch N/A, MOS Civilian. Participant 02 is male, age 35, height 1.80 meters, mass 96.1 kilograms, White, Army, Health Care Specialist. Participant 05 is male, age 29, height 1.83 meters, mass 90.9 kilograms, American Indian/Alaska Native, Army, Medical Laboratory Specialist. Participant 13 is female, age 26, height 1.55 meters, mass 67.8 kilograms, White/Black, Army, Animal Care Specialist. Branches represented include Army, Air Force, and Navy. MOS entries include Health Care Specialist, Medical Laboratory Specialist, Clinical Nurse, General Practice Physician, Orthopedic Cast Room Technician, Hospital Corpsman, Physical Therapist, Animal Care Specialist, and Medical Service Specialist. The last three rows provide summary statistics: Median age 30, height 1.78 meters, mass 89.9 kilograms; Maximum age 39, height 1.85 meters, mass 114.8 kilograms; Minimum age 25, height 1.52 meters, mass 56.0 kilograms. Abbreviations: AI/AN is American Indian/Alaska Native; MOS is Military Occupational Specialty. Mass was recorded during Visit 1 with the fitted vest and helmet.

Navigate back to Table 1..

## Figure 3. Long description

The top panel, labeled Medic, displays eight horizontal bars for subscales: Dimensions, Weight, Adjustability, Safety, Durability, Ease of Use, Comfort, and Effectiveness. Each bar is divided into five color-coded segments representing satisfaction levels from Not Satisfied at All to Very Satisfied. The x-axis shows Percentage of Respondents from 0 to 100. Mean subscale scores are listed at the right end of each bar: Dimensions 3.7, Weight 4.0, Adjustability 4.0, Safety 4.5, Durability 4.0, Ease of Use 4.2, Comfort 4.4, Effectiveness 4.4. The bottom panel, labeled Casualty, repeats the same subscales and format. Mean scores are: Dimensions 4.4, Weight 4.7, Adjustability 4.2, Safety 4.0, Durability 4.2, Ease of Use 4.0, Comfort 3.3, Effectiveness 4.2. In both panels, the majority of responses cluster in the Quite Satisfied and Very Satisfied categories, except for Comfort in the Casualty panel, which shows a higher proportion of lower satisfaction. The legend at the bottom defines the five satisfaction levels by color.

Navigate back to Figure 3..

## Figure 5. Long description

The top panel labeled Medic displays eight horizontal bars for Dimensions, Weight, Adjustability, Safety, Durability, Ease of Use, Comfort, and Effectiveness. Each bar is divided into five satisfaction levels: Not Satisfied at All, Not Very Satisfied, More or Less Satisfied, Quite Satisfied, and Very Satisfied, shown in increasing darkness of blue. The right side of each bar lists the mean subscale score: Dimensions 3.5, Weight 4.5, Adjustability 2.8, Safety 3.0, Durability 3.1, Ease of Use 3.0, Comfort 2.9, Effectiveness 3.0. Weight has the highest mean score and largest proportion of ‘Very Satisfied’ responses. The bottom panel labeled Casualty follows the same structure. Mean scores are: Dimensions 3.8, Weight 4.8, Adjustability 3.0, Safety 3.3, Durability 3.3, Ease of Use 3.3, Comfort 3.3, Effectiveness 4.0. Weight and Effectiveness have the highest mean scores and largest ‘Very Satisfied’ segments. The x-axis for both panels is Percentage of Respondents, ranging from 0 to 100. The legend at the bottom defines the five satisfaction categories by color.

Navigate back to Figure 5..
